# Comparative genomics and experimental promoter analysis reveal functional liver-specific elements in mammalian hepatic lipase genes

**DOI:** 10.1186/1471-2164-8-99

**Published:** 2007-04-11

**Authors:** Diederik van Deursen, Gert-Jan Botma, Hans Jansen, Adrie JM Verhoeven

**Affiliations:** 1 Department of Biochemistry, Cardiovascular Research School COEUR, Erasmus MC, PO Box 1738, 3000 DR Rotterdam, The Netherlands; 2 Department of Clinical Chemistry, Cardiovascular Research School COEUR, Erasmus MC, PO Box 1738, 3000 DR Rotterdam, The Netherlands

## Abstract

**Background:**

Mammalian hepatic lipase (HL) genes are transcribed almost exclusively in hepatocytes. The basis for this liver-restricted expression is not completely understood. We hypothesized that the responsible cis-acting elements are conserved among mammalian HL genes. To identify these elements, we made a genomic comparison of 30 kb of 5'-flanking region of the rat, mouse, rhesus monkey, and human HL genes. The in silico data were verified by promoter-reporter assays in transfected hepatoma HepG2 and non-hepatoma HeLa cells using serial 5'-deletions of the rat HL (-2287/+9) and human HL (-685/+13) promoter region.

**Results:**

Highly conserved elements were present at the proximal promoter region, and at 14 and 22 kb upstream of the transcriptional start site. Both of these upstream elements increased transcriptional activity of the human HL (-685/+13) promoter region 2–3 fold. Within the proximal HL promoter region, conserved clusters of transcription factor binding sites (TFBS) were identified at -240/-200 (module A), -80/-40 (module B), and -25/+5 (module C) by the rVista software. In HepG2 cells, modules B and C, but not module A, were important for basal transcription. Module B contains putative binding sites for hepatocyte nuclear factors HNF1α. In the presence of module B, transcription from the minimal HL promoter was increased 1.5–2 fold in HepG2 cells, but inhibited 2–4 fold in HeLa cells.

**Conclusion:**

Our data demonstrate that searching for conserved non-coding sequences by comparative genomics is a valuable tool in identifying candidate enhancer elements. With this approach, we found two putative enhancer elements in the far upstream region of the HL gene. In addition, we obtained evidence that the -80/-40 region of the HL gene is responsible for enhanced HL promoter activity in hepatoma cells, and for silencing HL promoter activity in non-liver cells.

## Background

Understanding transcriptional regulation of gene expression is a major challenge in molecular biology. In eukaryotes, regulation of gene expression is achieved through the complex interaction of transcription factors, which bind to specific DNA sequence motifs. These motifs are predominantly located in the upstream region of genes. Over the last decades, numerous transcription factors have been identified, each with its own specific DNA binding sequence (TFBS). Transcription factors that are potentially involved in the regulation of a particular gene are usually identified by the presence of the specific DNA binding motif in the upstream regulatory region. These binding motifs are compiled in libraries such as the Transfac database [1], and programs such as MatInspector enable pattern recognition with the entries in this database [2]. Unfortunately, most transcription factors bind to short, degenerate sequences, which occur very frequently in the eukaryotic genome. Only a very small fraction of all predicted binding sites is biologically relevant [3]. Recently, new strategies for the *ab initio *identification of functionally significant *cis*-acting regulatory sequences have been developed, based on the assumption that regulatory elements are conserved among multiple species [4-8], and that multiple TFBS tend to specifically cluster together [9,10]. The rVista computational tool for identification of functional regulatory elements combines the comparative sequence analysis of orthologous genes with the analysis of clustering of predicted TFBS [11,12]. In this study, we tested the validity of this approach to identify functional TFBS for the mammalian hepatic lipase genes, by comparing the *in silico *data with experimental promoter-reporter assays.

Hepatic lipases (HL) are synthesized and secreted almost exclusively by hepatocytes [13-15]. Although synthesis of HL has been shown to occur in mouse adrenals [16], and in mouse and human macrophages [17], this is negligible compared to expression in liver. The HL activity present in adrenals and ovaries [18] originates predominantly from liver, and is transported through the circulation to these organs [19,20]. In liver, the enzyme is bound to cell surface proteoglycans within the sinusoids, from where it can be released by heparin. Hepatic lipase plays an important role in plasma lipoprotein metabolism and intracellular lipid homeostasis [21], by mediating cholesterol influx into liver cells from high-density lipoproteins (HDL), and clearance of remnant lipoproteins from the circulation by the liver. HL is an important determinant of plasma HDL cholesterol levels, and is implicated in the protection against development of premature atherosclerosis by HDL [21]. HL gene expression in humans and rodents is regulated by various hormones and nutritional states mainly at the transcriptional level, but up- or downregulation is limited to about two-fold [15]. In contrast to this moderate regulation by hormones and nutrition, the almost complete restriction of HL gene expression to differentiated liver cells is highly conspicuous [13,14]. Several groups have pointed to the HNF1 and HNF4α binding sites in the proximal promoter of the HL gene to explain this liver-specificity in humans [22-25]. Since the liver-restricted expression is a common feature of most, if not all, mammalian HL genes, we hypothesize that the regulatory elements responsible for liver-specific expression are conserved among mammals. We therefore searched the upstream regulatory region of the rat, mouse, rhesus monkey and human genes for the presence of conserved clusters of TFBS motifs, and combined the *in silico *data with experimental promoter-reporter assays in cultured cells of hepatic versus non-hepatic origin. This unbiased approach led to the identification of two putative enhancer elements in the far upstream region, and of highly conserved sequence modules within the proximal promoter of the HL genes.

## Results

### Interspecies comparison of genomic HL sequences

**Figure 1:**
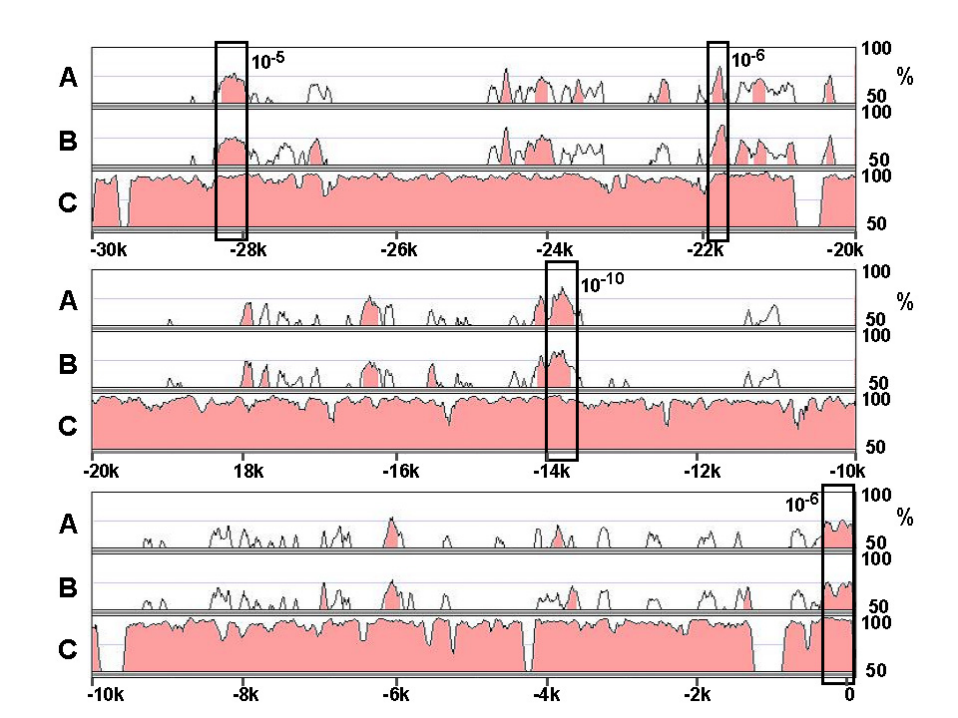
**Alignment of the 5'-flanking region of four mammalian HL genes **. From the rat, mouse, macaque and human HL genes, exon-1 and 30 kb of upstream sequence was aligned by the MLAGAN algorithm of the mVista program. The sequences of rat (A), mouse (B) and macaque (C) are aligned to the human HL sequence (x-axis); numbering is relative to the transcriptional start site. Conserved regions (> 70% homology over 100 bp window) are shaded. The boxes indicate conserved regions among the four sequences, as determined by RankVista (*P *≤ 10^-5^), with the P-values given above.

Of the mammalian HL genes, genome sequence including part of the 5'-flanking region is available for human, chimpanzee, rhesus monkey, rat, mouse and hedgehog (Ensembl ***e****!*42:Dec 2006)[26]. Pairwise alignment of the HL coding sequences shows the expected, high degree of sequence identity (Table 1). This high homology also extends into the 5'-UTR and upstream-regulatory region, with sequence identity ranging from 53 to 98 % over the proximal 0.9–1.4 kb. Multiple sequence alignment of 30-kb of the 5'-flanking region available for five HL genes (all except for the hedgehog, which known sequence is too short) was performed by the mVista web-tool (Fig. 1). The chimp and macaque sequences are highly homologous to the human sequence, as 95 % and 64 % of the 30-kb region showed at least 95 % sequence identity over a 100-bp window, respectively. Similarly, 5 % of the rat sequence showed this high degree of sequence identity with the orthologous mouse sequence. Because of the near-identity of the chimp to the human sequence, we only included the latter sequence in subsequent *in silico *analysis. The global genomic sequence comparison showed a particularly high conservation among the four genes immediately upstream of the transcriptional start site (*P *= 7*10^-7^). Three additional islands of highly significant homology (*P *< 10^-5^) were identified further upstream. Conservation of a 475 bp element at -14 kb was even more significant (*P *= 4*10^-11^) than the proximal HL promoter region (Fig. 1). The element at -22 kb (*P *= 2*10^-6^) contained a 173 bp sequence that was completely identical among the three primate sequences.

**Figure 2:**
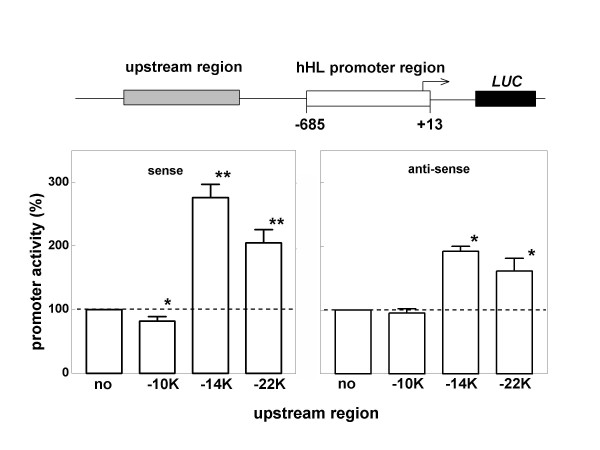
**Possible enhancer activity of the conserved sequences in the far upstream HL regulatory region**. HepG2 cells were transiently transfected with the indicated promoter-reporter constructs. At 48 h post-transfection, transcriptional activity was determined as the firefly over renilla luciferase activity. Data are expressed as percentage of the ratio measured in the hHL-685luc – transfected cells. Data are means ± SD from 3–4 independent experiments, each performed in quadruplicate. *and **: *P *< 0.05; and *P *< 0.01, respectively.

To test the potential enhancer function of two of the most conserved elements in the far upstream regulatory region, promoter-reporter assays were performed with human HL promoter constructs in transiently transfected HepG2 hepatoma cells. The -14 kb element (679 bp) and the -22 kb element (387 bp) were inserted in the hHL-685Luc plasmid. As a control, we also tested the activity of the non-conserved -10 kb sequence (502 bp). The transcriptional activity of the human HL -685/+13 promoter region was increased 3- and 2-fold with the -14 kb and -22 kb elements inserted in the sense orientation, respectively (Fig. 2). In contrast, the -10 kb sequence slightly but significantly reduced HL promoter activity (n = 3; *P *< 0.05). Qualitatively similar effects were obtained when the elements were inserted in the anti-sense orientation (Fig. 2). Hence, the conserved sequences at -14 kb and -22 kb have moderate enhancer activity of the proximal HL promoter region.

### Comparative genomics of the proximal 2 kb HL upstream regulatory region

**Figure 3:**
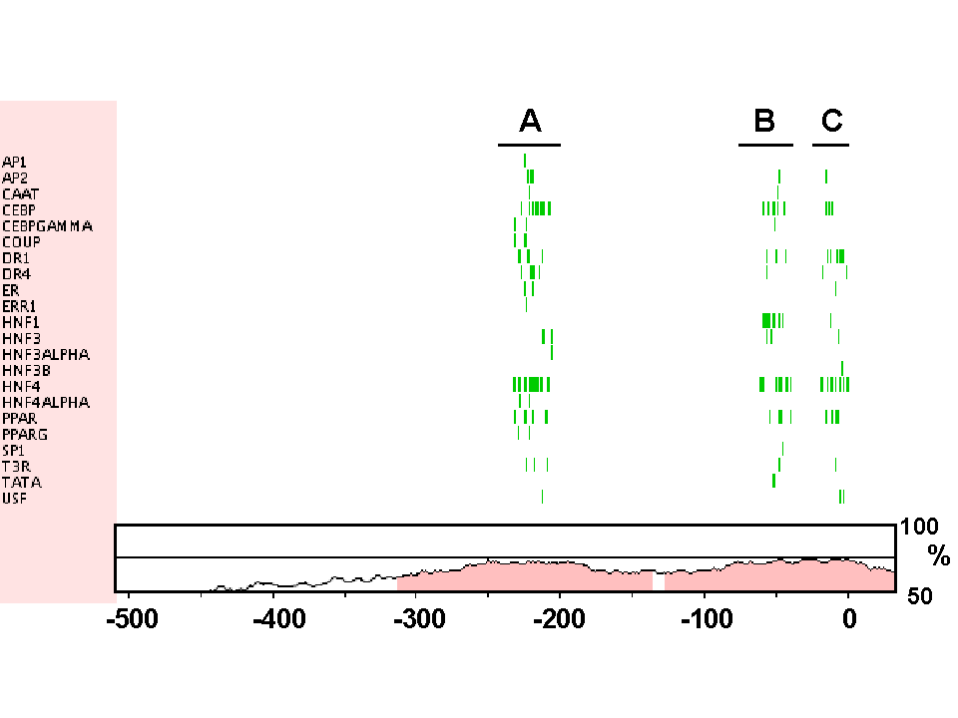
**Identification of functional regulatory sequences in the proximal promoter region of the HL gene by rVista**. Of the proximal promoter regions of the rat and human HL genes, 600 bp were submitted to the rVista sequence analysis software, and searched for conserved clusters of TFBS for a selection of 50 transcription factors known to be expressed in mammalian liver. A vertical line indicates the position of the conserved TFBS relative to the human sequence (x-axis, numbering is relative to the transcriptional start site). Homology between the rat and human sequence is given as described for figure 1. Three clusters of conserved TFBS are identified, and designated A (-240/-200), B (-80/-40) and C (-25/+5). In human-mouse comparison, these clusters are also evident with an additional cluster at -295/-265.

Submission of 2-kb upstream sequence of the rat HL gene to the MatInspector software program (core similarity > 0.75; matrix similarity > 0.70) returned over 2000 potential TFBS, randomly distributed over the entire sequence. A similar number of sites was predicted for the orthologous mouse, human and rhesus macaque sequences. When we searched for clustered TFBS motifs that are conserved between the rat and human sequence, using the web-tool rVista, three separate modules were identified within the proximal promoter region (Fig. 3). Module A (-240 to -200 in the human sequence relative to the transcriptional start site), for which AP1, AP2, CAAT, COUP, C/EBP, HNF4α, PPAR and USF binding sites are predicted, corresponds to the DR1 site recently identified by Rufibach *et al*. [25]. Module B (-80 to -40) potentially contains AP2, CAAT, C/EBP, HNF1, HNF4, PPAR and Sp1 sites, and corresponds to the previously characterized HNF1 site [22-24]. Module C (-25 to + 5), which may bind AP2, C/EPB, HNF4, PPAR and USF factors, contains the transcription start site preceded by a conserved pyrimidine-rich motif, and therefore likely represents the Inr involved in binding of the transcription initiation complex. These three modules were also found to be conserved among the human and mouse HL gene. The human-mouse comparison revealed an additional, conserved module (-295 to -265), with potential binding sites for AP2, C/EBP, HNF1, HNF4, PPAR and Sp1, and which partly overlaps the DR4-site recently described by Rufibach *et al*. [25]. Similar results were obtained in pairwise comparisons between orthologous sequences of macaque and rat, and of macaque and mouse. Despite the high homology in the intervening sequence between modules A and B, the rVista program did not recognize conserved clusters of TFBS among the human, macaque, rat and mouse. Irrespective of which transcription factors actually bind to these sites, the results of the interspecies sequence comparison by rVista suggest that the three highly conserved sequence modules in the proximal HL promoter region are involved in common features of transcriptional regulation. This is further supported by the fact that these three modules correspond to distinct DNA footprints of the human HL sequence in rat liver [22] and human HepG2 cells [23].

### Functional characterization of the rat HL promoter region

**Figure 4:**
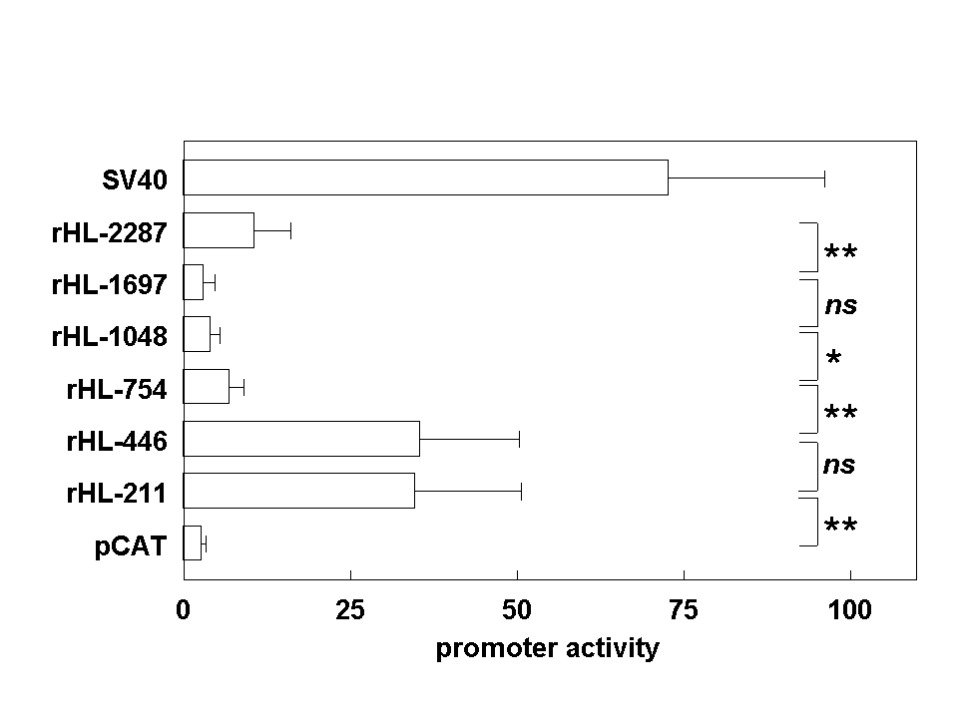
**Effect of serial 5'-deletions of the rat HL upstream region on transcriptional activity in HepG2 cells**. HepG2 cells were transiently transfected with the indicated promoter-reporter constructs. At 48 h post-transfection, cells were lysed and expression of CAT and β-galactosidase protein was determined. Data are expressed as the ratio of CAT over β-galactosidase expression. Data are means ± SD from 4–7 independent experiments, each performed in triplicate. *: *P *< 0.05; **: *P *< 0.01; n.s.: not significant.

To corroborate the *in silico *results, promoter-reporter assays were performed with promoter fragments of the rat HL gene in transiently transfected HepG2 cells. Plasmids were constructed with progressively 5'-deleted promoter fragments spanning the -2287/+9 region of the rat HL gene in front of the CAT reporter gene. Compared to the SV40 promoter, the rHL-2287 construct showed low CAT expression (Fig. 4). Upon deleting the 5'-end of the HL promoter fragments to position -1048, CAT expression became even lower, and was no longer significantly different from promoter-less pCAT-Basic, suggesting that there is weak enhancer activity between nucleotides -1697 and -2287. Further deletion to position -754 slightly increased promoter activity to levels significantly above background. Shortening the insert from -754 to -446 resulted in a 5-fold increase in promoter activity, suggesting the presence of a strong negative regulatory element in this region of the rat HL gene. CAT expression was not significantly affected by deleting the insert from -446 to -211. The presence of the weak enhancer element between -2287 and -1697, and the negative element between -754 and -446 corresponds to positive and negative elements in the human HL upstream regulatory region observed by Oka *et al*. [23]. We assume, therefore, that both these elements are present in homologous parts of the rat and human gene. Indeed, the global alignment of the four species by mVista detected homology at these parts of the gene (Fig. 1), but homology did not exceed the 70 % over 100 bp mark used as threshold in this analysis. Apparently, potentially important elements may be missed due to the high stringency of the conservation rule in the mVista program.

**Figure 5:**
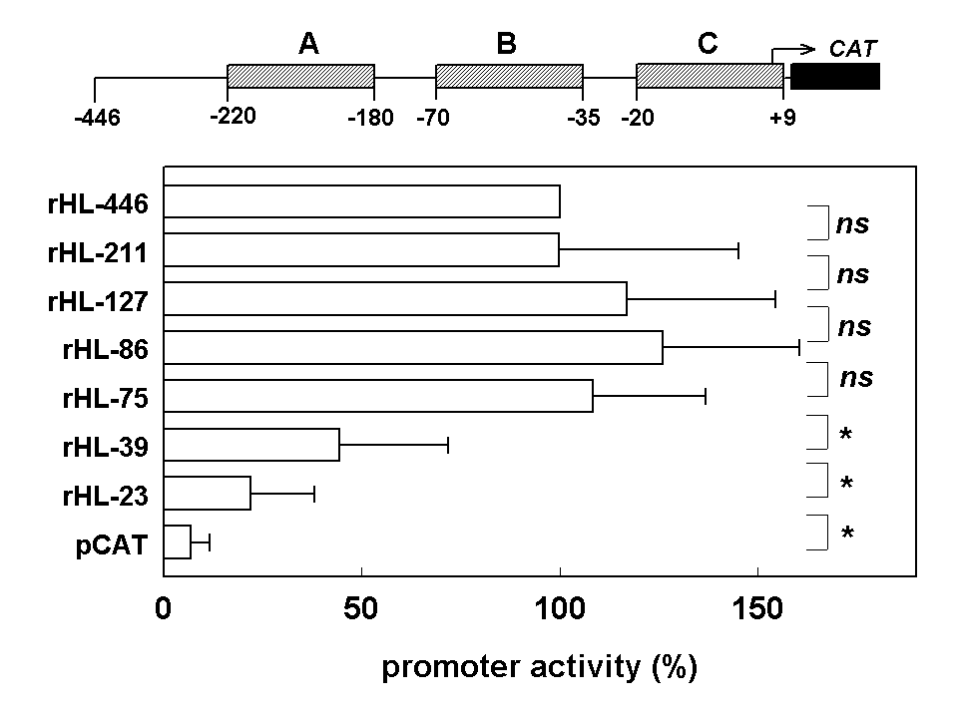
**Effect of serial 5'-deletions of the rat HL promoter region on transcriptional activity in HepG2 cells**. Experiments were performed as described in the legends to figure 4. Data were expressed as percentage of the ratio measured in the rHL-446 CAT – transfected cells, and are means ± SD from 3–5 independent experiments, each performed in triplicate. *: *P *< 0.05; n.s.: not significant.

To test the importance of the conserved sequence modules within the -220 to +9 region, further 5'-deletions in the rat HL promoter region were made (Fig. 5). Transcriptional activity of the rHL-127 construct, in which module A has been removed, was not significantly different from that of the rHL-446 or rHL-221 constructs. Similarly, removal of the highly conserved intervening sequence between modules A and B (rHL-86 and rHL-75) had no significant effect on CAT expression. In contrast, additional removal of most of module B in rHL-39 reduced transcriptional activity by approximately 60 %. With rHL-23, in which the remainder of module B as well as the putative TATA-box has been deleted, CAT expression decreased further. Despite absence of the TATA-box, CAT expression of the rHL-23 construct was significantly higher than of promoter-less pCAT-Basic, which may be due to residual promoter activity of module C.

### Comparison with the proximal human HL promoter region

**Figure 6:**
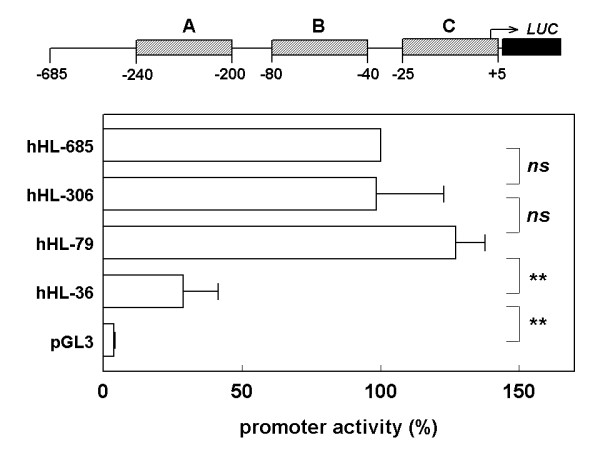
**Effect of serial 5'-deletions of the human HL promoter region on transcriptional activity in HepG2 cells**. HepG2 cells were transiently transfected with the indicated promoter-reporter constructs. At 48 h post-transfection, transcriptional activity was determined as the firefly over renilla luciferase activity. Data are expressed as percentage of the ratio measured in the hHL-685luc – transfected cells. Data are means ± SD from 4 independent experiments, each performed in quadruplicate. *: *P *< 0.05; n.s.: not significant.

Similar promoter-reporter assays were performed with the -685/+13 region of the orthologous human HL gene, except that the luciferase gene was used as reporter (Fig. 6). Luciferase activity of the hHL-306 construct was similar to hHL-685, whereas activity of the hHL-79 construct was slightly, but not significantly, higher. This is in line with the rat promoter data, which show little effect of module A, and of the intervening sequence between modules A and B, on basal transcriptional activity in HepG2 cells. The luciferase activity of the hHL-36 construct, in which entire module B has been removed, was only 25 % of the hHL-79 construct. The transcriptional activity of hHL-36, which contains a bona fide TATA box and entire module C, was 7-fold higher than background. This confirms that modules B and C are crucial for basal transcriptional activity in HepG2 cells, with module B being most important.

### Role of module B in liver cell-specific HL transcription

**Figure 7:**
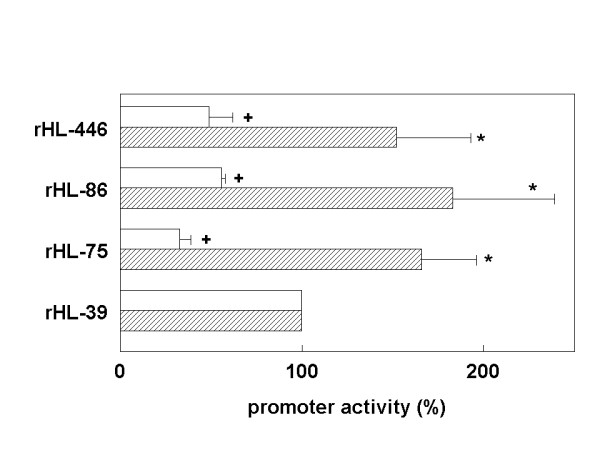
**Transcriptional activity of the rat proximal HL promoter region in HepG2 and HeLa cells**. HepG2 (hatched bars) and HeLa cells (open bars) were transiently transfected with the indicated promoter-reporter constructs. At 48 h post-transfection, transcriptional activity was determined as the ratio of CAT over β-galactosidase expression level. Data are expressed as percentage of the ratio measured in the rHL-39 CAT – transfected cells. Data are means ± SD from 3 independent experiments, each performed in triplicate. *,^+^: *P *< 0.05 compared to 100% in HepG2 and HeLa cells, respectively.

To test whether modules A and B are involved in liver-specific expression of the HL gene, we compared transcriptional activity of different rat HL promoter fragments in HepG2 cells with non-hepatic HeLa cells (Fig. 7). Promoter activity in each cell line was expressed as percentage of that of the rHL-39 construct, because this fragment represents the minimal promoter with the TATA-box and transcription start site. In the hepatoma cells, the activity of the rHL-75 construct was 1.5–2 fold higher than the minimal promoter construct. In HeLa cells, contrastingly, transcriptional activity of rHL-75 was 2–4 fold lower than the minimal promoter construct in HeLa cells. Consequently, there was a marked, 3–5-fold difference in relative promoter activity between these two cell lines. Similar results were obtained with the longer rat HL constructs that all contained module B. The data were minimally affected by the simultaneous presence of module A (Fig. 7). Qualitatively similar results were obtained with human HL promoter fragments (data not shown). We conclude therefore, that module B plays a pivotal role in liver-restricted expression of the HL gene, by moderately activating transcription in liver cells, and simultaneously suppressing activity in non-hepatic cells.

## Discussion

Global alignment of the 5'-flanking region of mammalian HL genes revealed three highly conserved elements (P ≤ 10^-5^) that lie far upstream of the HL promoter (Fig. 1). Two of these elements, at -14 kb and -22 kb, show moderate enhancer activity in HepG2 cells. What discriminates the conserved -14 kb and -22 kb elements from the non-functional, non-conserved -10 kb sequence is unclear, as all three sequences contain a similar repertoire of TFBS for liver-expressed transcription factors (data not shown). Further studies are required to clarify the mechanism responsible for the enhancer activity of the two highly conserved elements in the HL gene. The finding of two hitherto unknown enhancers supports the hypothesis that conserved non-coding sequences may identify functional regulatory elements. Experimentally, we also found a positive and a negative regulatory sequence between -2.2 and -0.4 kb of the rat HL gene that coincided with homology peaks, but were not recognized by the Rankvista analysis of the sequence comparison. Rubin's group recently demonstrated strong *in vivo *enhancer activity for almost half of the elements that are ultra-conserved among human/mouse/rat [8,27]. Our study further illustrates the power of the approach, and suggests that gene regulatory functions may also reside in somewhat less conserved elements among mammalian genomes.

We also tested whether global genome comparisons can also aid in identification of functional regulatory elements within highly conserved sequences, using the proximal HL promoter region as a model. Within this proximal promoter region, three modules are identified with conserved clusters of TFBS motifs. These modules A, B and C correspond with the previously identified regulatory elements DR1 [25], HNF1 [22-24] and Inr [22-24], respectively. However, we missed an additional module (-295 to -265) that has recently been identified as a functional DR4 site [25]. The cluster of TFBS within this module appeared to be conserved among human and mouse, but not among human and rat. Despite the relatively high homology between the mouse and rat over the proximal 5'-flanking region of the HL gene (Table 1), the outcome of the genomic sequence analysis differed whether the rat or the mouse sequence was used. Hence, although searching genomic sequences for conserved clusters of TFBS is a valuable tool in predicting functionally important regulatory elements, this approach is sub optimal.

For two of the modules that are conserved among the four species, a significant contribution to basal transcription was confirmed by promoter-assays in HepG2 cells. For module C (-25/+5), this is not surprising since it contains the transcriptional start site itself, as well as a pyrimidine-rich stretch that may serve as an initiator region (Inr). Module B (-80 to -40) overlaps with a protected region in DNAse footprinting in rat liver [22] as well as in HepG2 cells [23], and contains a HNF1 binding site that has been implicated in liver-specific expression of the human HL gene by other groups [22-24]. Experimentally, we could not confirm a major role for module A (-240 to -200) in determining basal transcription activity in HepG2 cells. This is surprising since it corresponds to a functional DR1 site [25], and perfectly matches with a protected region in DNAse footprinting in rat liver and human HepG2 nuclear extracts [22,23], suggesting that this part of the HL promoter is occupied by transcription factors under basal conditions. Similarly, we could not confirm the role of the DR4 module (-295 to -265) conserved among human and mouse, in basal transcriptional activity in HepG2 cells. We propose, therefore, that this part of the HL promoter region is involved in modulation of gene transcription under different hormonal or nutritional conditions.

We show here that the conserved module B (-80 to -40) plays a dual role in mediating liver-restricted transcription of the HL gene. On the one hand, the module mediates moderate stimulation of minimal promoter activity in liver-derived HepG2 cells, and on the other hand, it mediates inhibition of minimal promoter activity in the non-hepatic HeLa cells. Of the potential TFBS identified in module B, the liver-enriched HNF1 is a likely candidate for effecting the liver-specific activation of the HL promoter. Other groups have already suggested an important role for the HNF1 binding site [22-24], and in vitro HNF1 binding to this sequence has been demonstrated by gelshift assays [24]. Furthermore, HNF1α knockout mice have 3.4 fold lower HL mRNA levels than control mice [28]. In primary hepatocytes, HL secretion increases with HNF1α gene dosage [28]. However, HL mRNA and HL secretion are not completely lost by HNF1α knockout, indicating that HNF1α is not the only transcription factor determining HL expression in liver. HL secretion was only observed with hepatoma cell lines that express HNF1α or HNF1β mRNA [24], but not all cell lines with detectable HNF1α or -β expression do also secrete HL. In fact, HL secretion correlated with expression of HNF4 rather than with HNF1 mRNA [24]. The HNF4α gene itself is a target of HNF1α [29]. Since potential HNF4α binding sites were detected in the conserved module A (as well as in the -295/-265 module), the liver-specific stimulation of HL promoter activity may well be mediated by HNF4α. In fact, HNF4α is bound to the promoter regions of almost half of the actively transcribed genes in human liver [29] and therefore contributes to a large fraction of liver-specific gene expression. Sequence modules that contain both HNF1 and HNF4 binding sites are among the strongest predictors of liver-specific transcription [10]. Rufibach *et al*. [25] proposed that HNF1α and HNF4α independently and additively activate HL promoter activity. Which transcription factor(s) mediate inhibition of minimal promoter activity in cells of non-hepatic origin, remain(s) unknown at present.

## Conclusion

In summary, we have shown here that a global multispecies comparison of non-coding sequences, followed by a search for conserved clusters of TFBS, has predicted the most important sequences involved in basal transcription of the HL gene. This *in silico *analysis does not identify all regulatory sequences in a particular gene, but enables the intelligent design of experiments towards identification of functional cis-regulatory elements and transactivating factors in gene regulation. This study illustrates the power of comparative genomics in the identification of TFBS that are functional in gene expression.

## Methods

### Database analysis

The annotated data of the mammalian genome sequence projects were accessed through the Ensembl genome server (***e****!*42: Dec 2006) [30]. The exon-1 and 5'-upstream regulatory sequence of the hepatic lipase gene was available only for human (ENSG00000166035), rat (ENSRNOG00000015747), mouse (ENSMUSG00000032207), hedgehog (ENSETEG00000015177), chimpanzee (ENSPTRG00000007115) and rhesus macaque (ENSMMUG00000009566). Multiple sequence alignment was performed with DNAMAN software package version 3.2 (Lynnon BioSoft, Quebec, Canada). Global sequence alignments were performed with the publicly available web-based tool mVista [12,31] using the MLAGAN algorithm. A search for potential TFBS in the upstream regulatory region of a particular HL gene was performed online at Genomatix using the MatInspector program [2,32]. Clusters of TFBS that are conserved among the rat, mouse, human and macaque HL promoter regions were identified by the publicly available web-tool rVista [11,12,31].

### Isolation of exon-1 and the 5'-flanking region of the rat HL gene

A rat genomic library in λ DASH II (Stratagene, La Jolla, CA, USA) was used for isolation of the HL promoter region, using a HL cDNA probe corresponding to exons-1 and -2. The probe was generated by RT-PCR on 1 μg rat liver RNA using the oligonucleotides (5'-GGT AAG ACG AGA GAC ATG G-3', nt 1–19; numbering according to [33]) and (5'-CCC GTG GAT GAT CAT GAC AA-3', nt 285–266) as forward and reverse primers, respectively. The RT-PCR product was isolated by agarose gel electrophoresis, and radiolabeled using [α^32^-P]dCTP and the Megaprime kit from Amersham (Amersham, UK). Filters containing 10^6 ^plaques were hybridized overnight at 42°C with 50 ng of the labeled cDNA probe in hybridization buffer (50 % (v/v) formamide, 0.5 % (w/v) SDS, 0.1 mg/ml denaturated herring sperm DNA and 2 × PIPES buffer; [34]). After washing in 0.2 × sodium chloride/sodium citrate/0.5% SDS at 65°C for 5 min, the filters were exposed to autoradiography film. Two positive clones were identified, which were plaque-purified three times. One of these clones was selected for further analysis. Phage DNA was isolated and digested with *EcoR*I. A 6 kb fragment [35] was subcloned into pBluescript KS^- ^(pBsE6) and its identity with the 5'-regulatory region of the rat HL gene was verified by sequence analysis.

### Construction of reporter plasmids

The clone in pBluescript containing the 6 kb *EcoR*I fragment of the rat HL gene (pBsE6) was used to generate promoter-reporter constructs in pCAT-Basic (Promega, Madison, WI, USA). By digestion with *Pst*I and *Xba*I, a 1.85-kb *Pst*I/*Pst*I, a 0.32-kb *Pst*I/*Xba*I and a 0.15-kb *Xba*I/*Xba*I fragment was isolated. First, the 0.32-kb *Pst*I/*Xba*I (-446/-127; numbering according to [35]) fragment was cloned into pCAT-Basic. From this construct, the rHL-446 CAT plasmid was generated by insertion of the 0.15-kb *Xba*I (-127/+9) fragment. Subsequently, rHL-2287 CAT was generated by insertion of the 1.85-kb *Pst*I (-2287/-446) fragment into rHL-446. The rHL-127 CAT construct was made by subcloning the 0.15-kb *Xba*I (-127/+9) fragment into pCAT-Basic.

From the rHL-2287 CAT vector, the 5'-truncated rHL-1697, rHL-1041 and rHL-747 constructs were generated by PCR using *Hind*III-restriction site-containing oligonucleotides 3F, 4F and 5F as upstream primer, respectively, and the CAT-gene specific oligonucleotide CATrev2 as downstream-primer (Table 2). After digestion of the PCR products with *Hind*III and *Pst*I, the DNA fragments were purified by electrophoresis through agarose gel, and subsequently ligated into the rHL-446 CAT plasmid that had been digested with the same restriction enzymes. Similarly, the rHL-211 construct was generated from the rHL-446 CAT by PCR using oligonucleotides 9F and CATrev2 as upstream and downstream primer, respectively, followed by ligation into the *Hind*III and *Xba*I sites of rHL-446 CAT. Finally, the rHL-75, rHL-39 and rHL-23 constructs were generated from pBsE6 using 7F, 6F and 11F as upstream, and T3Primer as downstream primer, respectively, followed by digestion and ligation into the *Hind*III and *Pst*I sites of pCAT-Basic; subsequently, the resulting plasmids were digested with *Xba*I followed by self-ligation.

Human HL promoter constructs were made in the pGL3-Basic luciferase reporter plasmid (Promega, Madison, WI, USA), starting from the hHL(-685/+13)-CAT plasmid described previously [36]. An upstream *Sac*I restriction site was introduced by PCR using the HHL-685Sac primer (Table 2) and the downstream HHL+13Xba primer. After digestion with *Sac*I and *Xba*I, the gel-purified DNA products were ligated into the *Sac*I and *Nhe*I sites of pGL3, thus generating the hHL-685Luc plasmid. Similarly, hHL-306Luc, hHL-79Luc and hHL-36Luc plasmids were generated by using HHL-306Nhe, HHL-79Kpn, and HHL-36Kpn as upstream primers, respectively.

To test the enhancer activity of conserved upstream sequences, the -14 kb, -22 kb and -10 kb elements were inserted into the enhancer site of the hHL-685Luc plasmid using the *BamHI *and *SalI *restriction sites. Human genomic DNA was isolated from a buffy coat, and the -14 kb, -22 kb and -10 kb elements were PCR amplified using specific primers (Table 2). The PCR products were cloned into the pGEM T-easy vector (ProMega, Madison, WI, USA). After digestion of the plasmids with *BamHI *and *SalI*, the inserts were cloned into hHL-685Luc in either sense or anti-sense orientation.

All clones were verified by DNA sequencing using the Thermo-sequenase dye terminator kit (Amersham, UK) and the ABI 377 sequencer (Applied Biosystems, Foster City, CA, USA).

### Promoter reporter assays

HepG2 hepatoma cells and HeLa cells were cultured at 37°C and 5% CO_2 _in Dulbecco's modified Eagle's medium (ICN, Costa Mesa, CA, USA) supplemented with 10% (v/v) fetal calf serum (Gibco, Breda, Netherlands) and penicillin/streptomycin. Transfection of HepG2 cells with CAT-reporter constructs was performed by the calcium-phosphate co-precipitation method. At 24 h before transfection, the cells were plated in 6-wells plates at 20–30 % confluence. At 3 h before transfection, the medium was refreshed. Cells were co-transfected with 2.5 μg/well of the CAT reporter test plasmid and 0.2 μg/well of control RSV β-galactosidase expression plasmid (Promega) [36]. Parallel transfections with SV40-CAT-Control and empty pCAT-Basic plasmids were used as controls. Fourty-eight hours post-transfection, cell lysates were prepared. CAT and β-galactosidase were determined by ELISA (Roche). Promoter activity was expressed as pg CAT/ng β-galactosidase to correct for differences in cell number and transfection efficiency.

Transfections of HepG2 and HeLa cells with the luciferase-reporter constructs were performed in 24-wells plates with Lipofectamine Plus (Invitrogen, Groningen, Netherlands) using 0.4 μg of the luciferase-reporter construct and 20 ng of pRL-CMV (Promega) per well [37]. Cell extracts were prepared at 48 h post-transfection. The luciferase activity in the cell extracts was determined with the FireLight kit (Perkin-Elmer, Boston MA, USA) and the Packard Top Count NXT luminometer. Data were normalized for the Renilla activity measured in the same sample.

### Statistics

Experimental data are expressed as mean ± SD. Differences were tested for statistical significance by paired Student's *t*-test.

## List of abbreviations

CAT, chloramphenicol acetyltransferase; HDL, high-density lipoprotein; HL, hepatic lipase; HNF, hepatocyte nuclear factor; kb, kilo basepairs; TFBS, transcription factor binding sites

## Authors' contributions

DvD and GJB carried out the biochemical assays. HJ and AJMV conceived of the study and participated in its design and coordination. AJMV carried out the comparative genomic analysis, and drafted the manuscript. All authors read and approved the final manuscript.

## Figures and Tables

**Table 1:** Pairwise sequence comparison of the coding and the 5'-flanking sequence of mammalian HL genes.

	upstream regulatory sequence							
cDNA sequence	81		53	53	61	57	56	hedgehog^a^
	79	76		86	64	55	59	mouse
	79	76	92		63	57	57	rat
	85	82	79	79		94	94	rhesus^a^
	85	82	80	80	96		98	chimp
	85	82	79	79	96	99		human

	rabbit^b^	hedgehog	mouse	rat	rhesus	chimp	human	

Sequence identity (%) was determined by pairwise alignment using the DNAMAN software package (optimal alignment; gap open penalty 10.0, gap extension penalty 5.0). cDNA sequence was from the translation start ATG up till the stopcodon; the upstream regulatory region was from -1400 up till the translation start ATG.

^a^: Because of a gap in upstream regulatory region of the public sequence of the rhesus monkey and hedgehog HL gene (Ensembl ***e****!*42: Dec 2006), alignments with the rhesus and hedgehog sequence were done with the sequence from -900 and -1000 up till the start ATG, respectively.

^b^:the rabbit HL cDNA sequence was taken from [38].

**Table 2:** Oligonucleotides used to generate serial 5'-deletions of the rat and human HL promoter region.

Name	species and orientation^a^		position^b^	5'→3' sequence^c^
3F	r	F	-1697/-1675	cggaagc TTA GCA GAC AGC GAT TGG C
4F	r	F	-1048/-1030	cggaag CTT GCC TCC TCC TGA GTG C
5F	r	F	-754/-736	cggaagc TTG TCC AGG GCG TCC ATA C
9F	r	F	-211/-196	cggaagctt AGC TTG GCT CAA AAG G
8F	r	F	-86/-71	cggaagctt GTG TTC AAA TAC TGG G
7F	r	F	-75/-58	cggaagct TGG GTA ACA TGT TTT AGG
6F	r	F	-39/-22	cggaag CTT CCA CAA CTA AAT ACC
11F	r	F	-23/-8	cggaagctt CCA AGA AGC ATT CTG G
HHL-685Sac	h	F	-685/-667	ccgagctc TGG TCG CCT TTT CCC TAC C
HHL-306Nhe	h	F	-306/-291	gcatgctagc GAA GCC ACC TAC CCC G
HHL-79Kpn	h	F	-79/-55	ggggtacc TAA CAT GTT GAG AGG
HHL-36Kpn	h	F	-36/-20	ggggtac CAA AGT ATC TAA TAG GC
HHL+13Xba	h	R	+13/-6	gctctaga CTT GGT AAT TTC TGA AGC C
HHL-10Kfw	h	F	-10457/-10437	gtcgac GAA GGA TCA GGT GAG GGA TGG
HHL-10Krev	h	R	-9956/-9975	ggatcc GCT TCA AGG GCA ATG AAA GC
HHL-14Kfw	h	F	-14202/-14183	gtcgac GAC TTG GGG ATA TCC ACA TC
HHL-14Krev	h	R	-13524/-13549	ggatc CTG GTA AAA GGA CAT GAA CAA TAT GG
HHL-22Kfw	h	F	-21929/-21909	gtcgac GAT GGG AAA TGG ACC TAC AGC
HHL-22Krev	h	R	-21543/-21562	ggatcc GAT GAG GAC TGA TTC TCA GC
CATREV2^d^	-	R		gca act gac tga aat gcc tc
T3primer^e^	-	R		att aac cct cac taa ag

^a^: r and h: rat and human, respectively; F and R: forward and reverse orientation.

^b^: numbering of the rat and human sequence according to [35] and Ensembl ***e****!*42 [26], respectively.

^c^: rat and human HL specific parts of the primer sequences are given in capitals.

^d^: oligonucleotide specific for pCAT-Basic.

^e^: oligonucleotide specific for pBluescript.
